# An Orthotropic Elastic-Plastic Constitutive Model for Masonry Walls

**DOI:** 10.3390/ma13184064

**Published:** 2020-09-13

**Authors:** Piotr Bilko, Leszek Małyszko

**Affiliations:** Faculty of Geoengineering, University of Warmia and Mazury in Olsztyn, Oczapowskiego 2, 10-719 Olsztyn, Poland; leszek.malyszko@uwm.edu.pl

**Keywords:** orthotropic failure criteria, implementation, finite element, plasticity, masonry

## Abstract

The use of a continuum structural model for the analysis of masonry structures in the plane stress state is discussed in this paper. Attention is paid to orthotropic masonry at the material level and validation of the model after its implementation in a proprietary finite element method (FEM) system via user-supplied subroutine. The constitutive relations are established in the framework of the mathematical elastoplasticity theory of small displacements and deformations. Based on the orthotropic failure criterion that was originally proposed by Hoffman in the spatial stress state, the model includes a generalization of the criterion in the plane stress. As it is the case for isotropic quasi-brittle materials, different yield surfaces are considered for tension and compression, which are both of Hoffman type.

## 1. Introduction

The mechanical behavior of masonry is subjected to various influencing factors, mostly resulting from its complicated mesoscopic and microscopic structure and two basic materials used. Therefore, different modeling approaches are available for the numerical simulation of the mechanical behavior of masonry structures. Extensive research has been conducted on the development of advanced numerical modeling and the analysis of historical masonry structures for several decades [1,2,3,4]. However, a macroscopic approach is required for the viable analysis and the prediction of global structural behavior. The macroscopic composite behavior of masonry can be described assuming a homogeneous continuum and an anisotropic material with directional properties. This alternative of the constitutive modeling proved to be promising in two-dimensional problems, especially for models with closed form of the failure, yield, or limit surface. Some constitutive macro-models that are relied on for the finite element method (FEM) are widely used in the last decades with a different degree of complexity and idealizations—from initial models with simply isotropic, linear elastic behavior to advanced models with non-linear orthotropic behavior that are recently developed in the framework of modern concepts of continuum damage mechanics.

More considerable interest in the biaxially loaded masonry began more than forty years ago, in the late seventies of the last century, both by experiments and theory. In general, experiments were mainly carried out at that time concerning the failure of shear walls. They were usually related to the proposed failure criterion for the masonry in the plane state of stresses in the representative form of the particular tests that had to be involved [5,6]. The representative biaxial test for walls built with any kind of masonry elements was conducted very seldom because of the technical difficulties. The biaxial test data of Page [7,8] were interpolated in [9] in the form of a failure criterion. The criterion was written using three failure surfaces in the form of elliptic cones expressed by a second-order polynomial of stress components in the reference axes coaxial with masonry layers and without any reference to the observed distinct failure modes of masonry panels. On the other hand, an important composite failure criterion which was derived based on the four different failure mechanisms was proposed in [10]. This criterion was next used in the FEM-based constitutive model in [11].

There have been few attempts to use a single failure surface in constitutive models of masonry panels because of the non-acceptable fit of experimental values. The general orthotropic failure criterion of Tsai-Wu [12] was already available for composite materials since 1971. The use of this criterion for masonry was attempted in [13] in the form of a polynomial function of the second degree in stresses. A criterion in the form of a double pyramid with a rectangular base and the slope angle equal to the internal friction angle of the material was assumed in [14]. The non-acceptable fit of Page’s experimental values resulting from the Hoffman [15] single surface criterion was discussed in [16], although the criterion itself as a single limit condition is quite flexible and attractive to use. The phenomenological single-surface constitutive model cannot also distinguish between different failure modes. In the framework of computational plasticity, the use of a composite yield criterion containing several failure criteria seems to overcome this drawback. The choice of two failure surfaces, one for the tension regime and the second one for the compression regime, provides better agreement with Page’s experimental results. To describe the orthotropic behavior of masonry, two orthotropic yield criteria were used in [17], where a hydrostatic pressure insensitive material of a Hill-type as the yield criterion for compression together with a Rankine-type yield criterion for tension were assumed. The composite criterion containing three failure surfaces, each of them being for tension, compression, and shear regimes, allows distinguishing between different failure modes [18]. Contours consisting of a few surfaces are characterized good compatibility with the experimental results—see, e.g., [19,20] and [18,21]. However, this approach seems to be too demanding a computational task of plasticity.

Concerning masonry inelastic behavior, the closed-form macro-models are more efficient and suitable for complex structural computations. Some constitutive models that are recently developed in the framework of modern concepts of continuum damage mechanics are based on the assumption that the masonry axes of the bed and head joints are also damage principal axes. Usually, scalar damage parameters are assumed in each direction of the fixed axis, see for instance [14], where two independent scalar parameters in each direction of the material axis were used and their evolution is described by the energy-based approach. A similar approach in the framework of computational plasticity was used in [17], where the principal directions of damage are fixed and aligned with the initial orthotropy axes and softening/hardening relationships were adopted for the stress–strain diagrams in tension and compression, with different fracture energies along the axes of each material. The energies were coupled by a single scalar internal parameter used in the plasticity algorithm to measure simultaneously the amount of softening/hardening in two material axes. The main drawback of the closed-form orthotropic macro-models is the identification of the material parameters. To estimate macro-scale properties from mortar and brick parameters and their bonding, homogenization techniques can be used, both in elastic as well as plastic behavior, e.g., [22,23,24]. An alternative option is to transfer the identification problem to the level of masonry constituents by using multi-scale methods, e.g., [18,20,21], or to use methods developed recently for modeling of anisotropic quasi-brittle fracture, e.g., the so-called phase-field fracture model in the diffusive damage mechanics [25,26,27].

A continuum damage model in which the orthotropic behavior is simulated using a mapping relationship between the orthotropic behavior and an auxiliary model has been recently published in [28]. By using the concept of the mapped stress tensor the problem can be more efficiently solved in the mapped space and the results can be transported to the real field. Two distinct isotropic failure criteria are assumed in the mapped space and two stress transformation tensors are adopted. In the paper, the computational representation of complex failure loci obtained by experiments on orthotropic masonry is also presented.

This paper discusses an extension of the approach presented in [17]. Although the extension can be done both in a plane stress state as well as in a spatial stress state, the constitutive model with the generalization of the Hoffman criterion in a plane stress state is discussed here. Two orthotropic failure criteria are used, which are formulated in the framework of the representation theory of orthotropic tensor functions based on the Hoffman criterion [15]. The use of two Hoffman-type failure criteria as the yield criteria in the plasticity model seems to be particularly attractive and may give the better fit of the experimental values [29,30]. The composite masonry is treated as a homogenized orthotropic continuum. Since the failure criteria are scalar-valued functions of the stress tensor, the invariant representations of these criteria are dependent only on orthotropic invariants of the stress tensors. It is also the purpose of the paper to show the possibility of formulating robust numerical algorithms of the model implementation into a commercial finite element code at the integration point level using user-defined subroutines. Some tests of the proposed numerical algorithm for an anisotropic continuum are presented in the paper, both at the single element level and at the structural level and in the plane stress state.

## 2. The Orthotropic Hoffman-Type Failure Criteria in an Invariant Form

**Figure 1:**
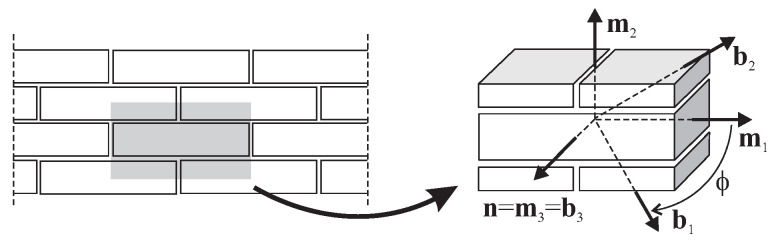
Axes of orthotropy $\left\{m_{i}\right\}$ and a Cartesian system $\left\{b_{i}\right\}$.

To model such effects as a marked difference observed between strengths in tension and compression, Hoffman [15] proposed a fracture criterion for brittle orthotropic materials as an extension to the Hill yield criterion. The criterion was proposed in the spatial stress state and in the $\left\{m_{i}\right\}$ frame of reference that coincides with the axes of orthotropy. The criterion was originally described by the function with nine material constants $C_{i}$ that are dependent on the three uniaxial tensile strengths $Y_{ti}$ and the three uniaxial compressive strengths $Y_{ci}$, along with the orthotropy directions *i* and also on the three shearing strengths $k_{ij}$ on the planes of material orthotropy. In the case of the plane stress state, when the normal vector to the stress plane is coincided with the axis of orthotropy $n=m_{3}=b_{3}$ (Figure 1), stress components $\sigma_{33}=\sigma_{31}=\sigma_{23}=0$ and the Hoffman criterion:

(1) $$\begin{array}{c} C_{1}{\left(\sigma_{22}-\sigma_{33}\right)}^{2}+C_{2}{\left(\sigma_{33}-\sigma_{11}\right)}^{2}+C_{3}{\left(\sigma_{11}-\sigma_{22}\right)}^{2}+\\ C_{4}\;\sigma_{11}+C_{5}\;\sigma_{22}+C_{6}\;\sigma_{33}+C_{7}\;\sigma_{23}^{2}+C_{8}\;\sigma_{13}^{2}+C_{9}\;\sigma_{12}^{2}-1=0\;, \end{array}$$

where:

(2) $$\begin{array}{c} C_{1}=\frac{1}{2}\left(\frac{1}{Y_{t2}Y_{c2}}+\frac{1}{Y_{t3}Y_{c3}}-\frac{1}{Y_{t1}Y_{c1}}\right),\;C_{2}=\frac{1}{2}\left(\frac{1}{Y_{t3}Y_{c3}}+\frac{1}{Y_{t1}Y_{c1}}-\frac{1}{Y_{t2}Y_{c2}}\right),\\ C_{3}=\frac{1}{2}\left(\frac{1}{Y_{t1}Y_{c1}}+\frac{1}{Y_{t2}Y_{c2}}-\frac{1}{Y_{t3}Y_{c3}}\right),\;C_{4}=\frac{1}{Y_{t1}}-\frac{1}{Y_{c1}}\;,\;C_{5}=\frac{1}{Y_{t2}}-\frac{1}{Y_{c2}},\\ C_{6}=\frac{1}{Y_{t3}}-\frac{1}{Y_{c3}}\;,\;C_{7}=\frac{1}{k_{23}^{2}}\;,\;C_{8}=\frac{1}{k_{31}^{2}}\;,\;C_{9}=\frac{1}{k_{12}^{2}}\;, \end{array}$$

takes the following form with the six material constants:

(3) $$C_{1}{\left(\sigma_{22}\right)}^{2}+C_{2}{\left(\sigma_{11}\right)}^{2}+C_{3}{\left(\sigma_{11}-\sigma_{22}\right)}^{2}+C_{4}\;\sigma_{11}+C_{5}\;\sigma_{22}+C_{9}\;\sigma_{12}^{2}-1=0\;,$$

where the constants $C_{1}÷C_{3}$ are dependent on uniaxial strengths $Y_{t3}$ and $Y_{c3}$ in the direction perpendicular to the stress plane.

The theory of tensor functions together with the theorems on their representations has been recognized to be an efficient mathematical tool for the formulation of constitutive relationships with both the desirable analytical clarity and required generality.

For some other recent applications of tensor functions see, e.g., [31,32]. It also allows accounting straightforwardly the invariance requirements of the principle of the space isotropy and the material symmetries so that the orientation of the material in space does not affect on its constitutive relation. Using this theory with Boehler’s results [33], we can assume that the orthotropic criterion (3) is a particular case of the more general scalar-valued orthotropic function of three invariants $tr\left(M_{1}\sigma \right)$, $tr\left(M_{2}\sigma \right)$, $tr\sigma^{2}$ of the following form:

(4) $$f\left(tr\left(M_{1}\sigma \right),tr\left(M_{2}\sigma \right),tr\sigma^{2}\right)-1=0\;,$$

where σ is the symmetric plane stress tensor ($\sigma \in T_{2}^{s},dimT_{2}^{s}=3$) and $M_{\alpha }$ are the parametric (structural) tensors defined as:

(5) $$M_{1}=m_{1}⊗m_{1},\;M_{2}=m_{2}⊗m_{2}.$$

The unit vectors $m_{\alpha }$ are the privileged directions of the orthotropic material, so they have to be perpendicular to each other. The invariants are very useful for the interpretation of the failure surface in any coordinate systems of the plane stress tensor that are different from the principal axes of orthotropy. Following the paper [34] or [35], we can choose another set of invariants $K_{p}$ in the form:

(6) $$K_{1}=tr\left(M_{1}\sigma \right),\;\;K_{2}=tr\left(M_{2}\sigma \right),\;\;K_{3}=tr\sigma^{2}-{\left(tr\left(M_{1}\sigma \right)\right)}^{2}-{\left(tr\left(M_{2}\sigma \right)\right)}^{2},$$

where the symbol “tr” denotes the trace of a second order tensor ($tr\left(\mathrm{AB}\right)=A_{ij}B_{ij}$). The form (6) is very convenient because in the $\left\{m_{\alpha }\right\}$ axes the invariants are:

(7) $$K_{1}=\sigma_{11},\;K_{2}=\sigma_{22},\;K_{3}={\left(\sqrt{2}\sigma_{12}\right)}^{\;2}.$$

Using the invariants (7), the criterion (3) can be treated as a particular case of the criterion proposed in [34] and may be written in the following invariant form:

(8) $$f\left(K_{i}\right)-1=a_{\alpha }K_{\alpha }+b_{\alpha \beta }K_{\alpha }K_{\beta }+cK_{3}-1=0,$$

where α,β=1,2, i=1,2,3 and the material constants are defined as:

(9) $$\begin{array}{c} a_{\alpha }=\frac{1}{Y_{t\alpha }}-\frac{1}{Y_{c\alpha }}\;,\;\;b_{11}=\frac{1}{Y_{t1}Y_{c1}}\;,\;\;b_{22}=\frac{1}{Y_{t2}Y_{c2}},\\ b_{12}=\frac{1}{2}\left(\frac{1}{Y_{t3}Y_{c3}}-\frac{1}{Y_{t1}Y_{c1}}-\frac{1}{Y_{t2}Y_{c2}}\right)\;,\;\;c=\frac{1}{2k_{12}^{2}}\;. \end{array}$$

Note that in the constant $b_{12}$ there are again uniaxial strengths $Y_{t3}$ and $Y_{c3}$ in the direction perpendicular to the stress plane.

The criterion (8) can be also written in the following invariant form:

(10) $$\frac{1}{2}\sigma \cdot P\cdot \sigma +p\cdot \sigma -1=0\;,$$

where a dot means a double contraction of two tensors, p is the symmetric tensor function of the second-order:

(11) $$p=a_{1}M_{1}+a_{2}M_{2},$$

P is the double symmetric tensor function of the fourth-order:

(12) $$P=2b_{11}M_{1}⊗M_{1}+2b_{22}M_{2}⊗M_{2}+2b_{12}\left(m_{1}⊗m_{2}+m_{2}⊗m_{1}\right)+cM$$

with the fourth-order tensor M:

(13) $$M=4\;N⊗N\;,\;N=\frac{1}{2}\left(m_{1}⊗m_{2}+m_{2}⊗m_{1}\right).$$

Several criteria proposed in the literature for orthotropic materials are special cases of the quadratic limit surface (8), including an elliptic failure surface according to Tsai and Wu [12] and criteria discussed recently in [30]. However, a phenomenological single-surface model may give an insufficient description of the mechanical behavior. It does not permit easy identification of failure modes and thus renders the description of different post-failure mechanisms very difficult. At least two failure criteria should be taken into consideration, the one for the compression regime and the second for tension regime. Each of them may be of the form (8) as proposed in [34] where the failure criterion for orthotropic materials in the spatial stress state is represented by two quadratic functions of the six invariants of the stress tensor and parametric tensors. It may more accurately describe the failure data distribution than classical limit surfaces, although it may include fifteen independent material parameters for the description of failure surfaces. On the other hand, the concept of a smooth single-surface description seems to be attractive from a numerical point of view and also allows for modeling of different inelastic behavior by changing size, shape, and location of a quadratic state function according to the form (8) in orthotropic stress space.

It is possible to propose a generalization of the Hoffman criterion for the orthotropic material in the plane state of stresses. The determination of the six material parameters in the criterion (3) requires six strength tests. The five standard tests are uniaxial loading along the axes of orthotropy (two tests for tension and two for compression) and the shearing test in the plane of stresses. If the test of the uniform biaxial compression is used for determining the strength $Y_{cc}$ as the sixth test in addition to conventional tests, we will get the criterion in the invariant form (8) or (10), in which the material parameter $b_{12}$ is changed to the form:

(14) $$b_{12}^{\left(1\right)}=\frac{1}{2Y_{cc}^{2}}-\frac{1}{2Y_{t1}Y_{c1}}-\frac{1}{2Y_{t2}Y_{c2}}+\frac{1}{2Y_{cc}}\left(\frac{1}{Y_{t1}}+\frac{1}{Y_{t2}}-\frac{1}{Y_{c1}}-\frac{1}{Y_{c2}}\right).$$

If as the additional test we will use the test of the uniform biaxial tension for determining the strength $Y_{tt}$, we will get the criterion in the invariant form with the following material parameter $b_{12}$:

(15) $$b_{12}^{\left(2\right)}=\frac{1}{2Y_{tt}^{2}}-\frac{1}{2Y_{t1}Y_{c1}}-\frac{1}{2Y_{t2}Y_{c2}}-\frac{1}{2Y_{tt}}\left(\frac{1}{Y_{t1}}+\frac{1}{Y_{t2}}-\frac{1}{Y_{c1}}-\frac{1}{Y_{c2}}\right).$$

Note that now there are not the uniaxial strengths $Y_{t3}$ and $Y_{c3}$ in the material constant $b_{12}$. Finally, using two functions of the form (8) or (10), one with the parameter (14) for compression regime $f_{1}\left(K_{i}\right)-1=0$ and the second with the parameter (15) for tension regime $f_{2}\left(K_{i}\right)-1=0$, we can construct the composite failure criterion in such a way that the following set:

(16) $$B=B_{1}\cap B_{2}\;\wedge \;B_{\alpha }\equiv \left\{\sigma \in T_{2}^{s}|\;f_{\alpha }\left(K_{i}\right)-1<0\right\},$$

is convex and

(17) $$f_{\alpha }\left(K_{i}\right)-1=a_{\alpha }^{\left(\alpha \right)}K_{\alpha }+b_{\alpha \beta }^{\left(\alpha \right)}K_{\alpha }K_{\beta }+c^{\left(\alpha \right)}K_{3}-1=0,$$

We can also assume that $c^{\left(1\right)}=c^{\left(2\right)}\equiv c$ and $a_{\alpha }^{\left(\alpha \right)}=a_{\alpha }$, $b_{\alpha \alpha }^{\left(\alpha \right)}=b_{\alpha \alpha }$ for simplification and we then have the following seven material parameters: the two uniaxial tensile strengths $\left(Y_{t\alpha }>0\right)$, the two uniaxial compressive strengths $\left(Y_{c\alpha }>0\right)$, the pure shear strength $\left(k_{12}>0\right)$, the biaxial uniform compressive strength $Y_{cc}>0$ and the biaxial uniform tensile strength $Y_{tt}>0$. This procedure is proposed in the paper [34], although the number of material parameters may be increased to 12 even though some of them can be used just to adjust failure surfaces to experimental data.

**Figure 2:**
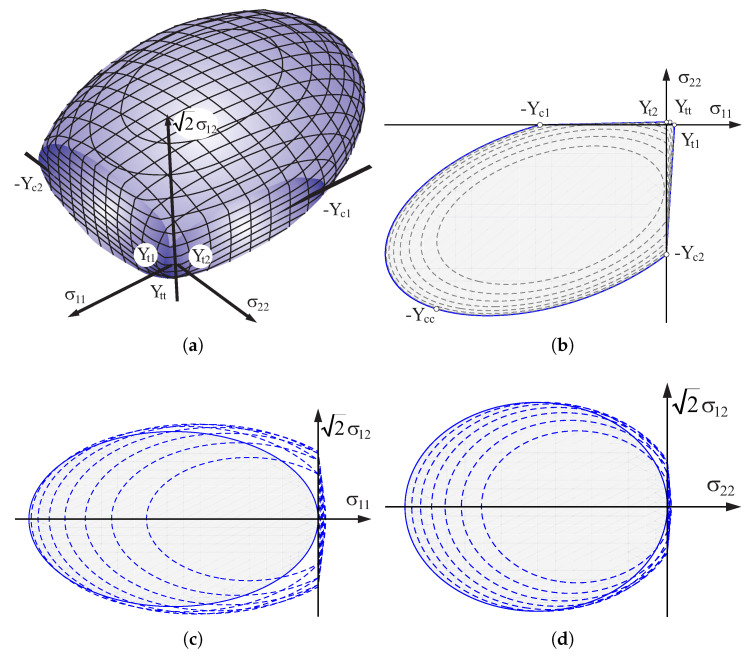
Criterion in orthotropy axes: (**a**) 3D view, (**b**–**d**) contours (described in text).

Figure 2 shows the criterion in the orthotropy axes with the following parameters for material: simple shear strength k=0.4 [MPa], vertical compression strength $Y_{c2}=6.34\;$ [MPa] and vertical tensile strength $Y_{t1}=0.31\;$ [MPa] perpendicular to the horizontal joint, compression strength $Y_{c1}=4.95\;$ [MPa] and tensile strength $Y_{t2}=0.14\;$ [MPa] parallel to the horizontal joint, biaxial uniform compression strength $Y_{cc}=9.0\;$ [MPa] and biaxial uniform tension $Y_{tt}=0.14\;$ [MPa]. Figure 2a shows general view and Figure 2b cross section of $\sigma_{12}=0$ (solid line) with contours every 0.2 [MPa] (dashed lines). Figure 2c shows cross section of $\sigma_{22}=-6.34\;$ [MPa] (solid line) with parallel contours every 0.81 [MPa] (dashed lines). Figure 2d shows cross section of $\sigma_{11}=-4.95\;$ [MPa] (solid line) with parallel contours every 0.81 [MPa] (dashed lines).

**Figure 3:**
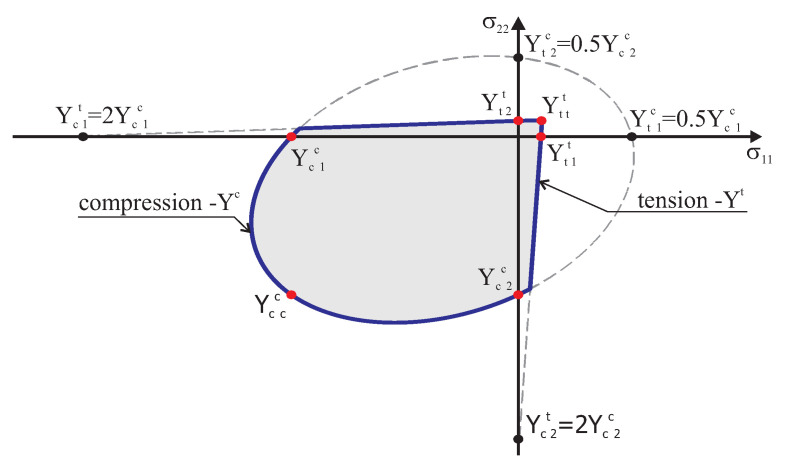
The proposed criterion and material parameters determining the surfaces.

An alternative connection of two surfaces is proposed, different from that used in [34], which is shown schematically in Figure 3. It allows for shifting a common edge. Figure 3 shows the method for constructing a boundary surface of the criterion in the normal stress components, in the orthotropy axes (at zero shear stresses). The surface in the tension range conventionally adopts compressive strength values $Y_{c\alpha }^{t}$ greater than twice those for the area of the compression range $Y_{c\alpha }^{c}$, that is $Y_{c\alpha }^{t}=2\;Y_{c\alpha }^{c}$. On the other hand, the surface in the compression range conventionally adopts tensile strength $Y_{t\alpha }^{c}$, by making them the value of compressive strength $Y_{c\alpha }^{c}$. It is assumed that they will be two-fold smaller, that is $Y_{t\alpha }^{c}=0.5\;Y_{c\alpha }^{c}$.

## 3. Comparison with the Experimental Results

**Figure 4:**
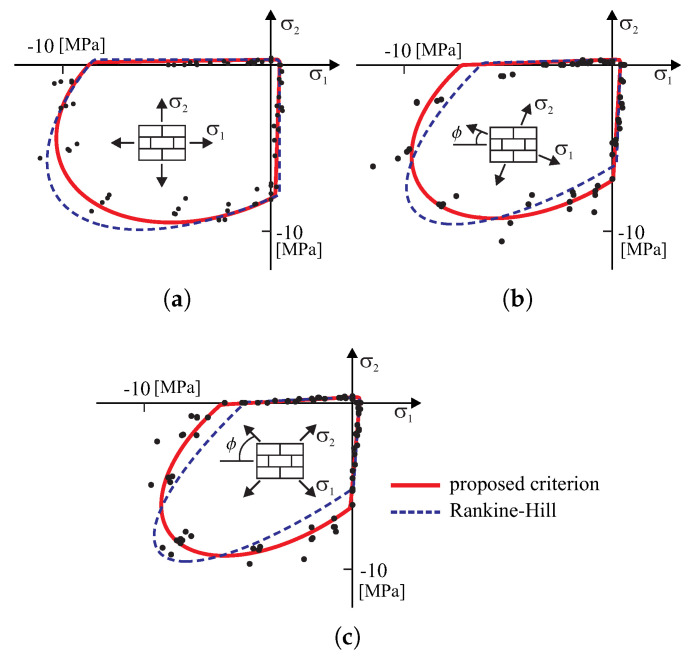
Comparison of the proposed criterion with the experimental results of Page [7,8] depending on the inclination axes of orthotropy relative to the principal axis: (**a**) ϕ=0.0, (**b**) ϕ=22.5, (**c**) ϕ=45.0.

The composite failure criterion is shown in Figure 4 in the orthotropy axes and in comparison with one of the most complete sets of experimental data of biaxially loaded masonry that was given by Page [7,8], who tested 102 panels of half-scale solid clay brick masonry. Tested elements were made on a scale of 1:2 with dimensions 360 × 360 × 50 [mm]. Tests were differentiated due to the rotation of the principal stress terms of the horizontal joint (axis of the material). The ratio of the principal stresses was changed so that the wall was considered in any possible state of stress. The following values of the parameters are adopted: axial tensile strength along the horizontal joint $Y_{t1}=0.43\;$ [MPa] and perpendicular to it $Y_{t2}=0.32\;$ [MPa], the axial compression strength along the horizontal joint $Y_{c1}=8.74\;$ [MPa] and perpendicular to it $Y_{c2}=8.03\;$ [MPa], the pure shear strength k=0.33 [MPa], uniform biaxial tensile strength $Y_{tt}=0.32\;$ [MPa] and compression strength $Y_{cc}=8.38\;$ [MPa]. Good agreement was found in the shape of the failure surface for principal stresses, which may mean that the criterion would appear to be sufficiently well validated for further investigations. Finally, the Rankine–Hill failure criterion [16] is shown in Figure 4 by dashed lines as a comparison to the proposed criterion.

Another discussed example is the comparison of the proposed failure criterion with the results of the experimental research from work [36]. The research program was carried out at the ETH Polytechnic in Zurich. Ganz and Thürliman’s team investigated biaxially loaded wall panels (designated K1–K12) with dimensions of 1200 × 1200 × 150 [mm${}^{3}$] and with different orientation of material axes to the principal stress directions. The results of experimental tests [36] are presented in Table 1 excluding two tests (K5 and K9), which concerned samples of reinforced walls. The second column of the Table 1 contains the proportion of stresses, while the third column gives the angle by which the system of material axes has been rotated to the load directions. The ratio of principal stresses allows the direction of the load path to be determined. The intersection point of the load path direction with the criterion surface determines the stress state, the components of which are placed in columns 7–9 of the Table 1. The criterion surfaces are determined by the following parameters [MPa]: for a tension regime—$Y_{t1}=0.28$, $Y_{t2}=0.01$, $Y_{c1}=3.74$, $Y_{c2}=15.72$, $k_{t}=0.048$, $Y_{tt}=0.01$ and for a compression regime—$Y_{t1}=0.94$, $Y_{t2}=3.81$, $Y_{c1}=1.87$, $Y_{c2}=7.61$, $k_{c}=2.868$, $Y_{cc}=2.06$. The anisotropy ratio of the compressive strength is $Y_{c2}/Y_{c1}=4.07$ and is related to the arrangement of cores in ceramic masonry elements. The length of the load vector should be determined as $\sqrt{\sigma_{11}^{2}+\sigma_{22}^{2}+2\sigma_{12}^{2}}$. The last column of the Table 1 presents the values of the ratios of the length of the vectors resulting from the experiment and from the initial surfaces of the proposed criterion. The results show good compliance of the criterion with the experimental results.

**Figure 5:**
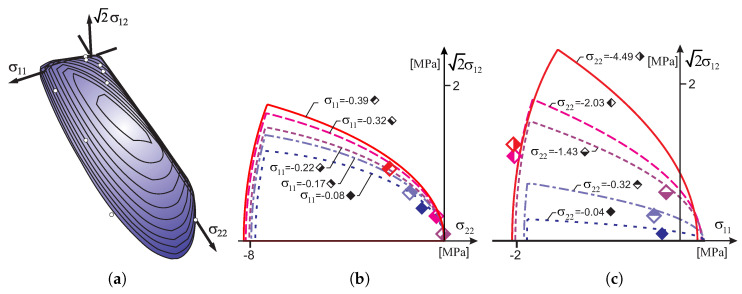
Comparison of the proposed base contour of the failure surface with the experimental results of Ganz and Thürliman [36]: (**a**) 3D view, (**b**) by cross sections of the body with $\sigma_{22}=const$, (**c**) by cross sections of the body with $\sigma_{11}=const$.

Figure 5a shows the initial surface of the criterion with points marked on the surface, indicating the stress limits of individual experimental tests conducted by Ganz and Thürliman. Analyzing the contour lines, it can be seen that most of the safe area is limited by the tensile regime. This is more clearly seen in Figure 5b,c, in which the plane cross-sections of the criterion are shown. One can see cross-sections with assigned to them, the values of the ultimate stress obtained from the individual experimental tests which are marked with diamonds.

**Figure 6:**
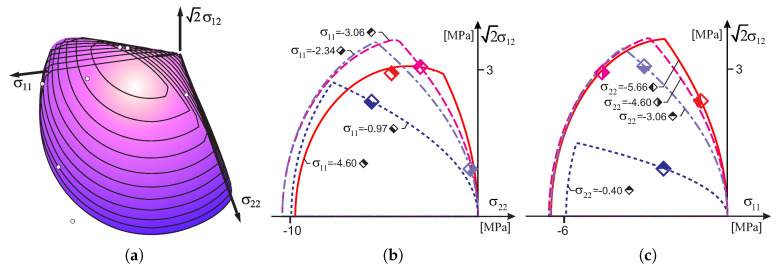
Comparison of the proposed criterion with the experimental results of of Lurati [37]: (**a**) 3D view, (**b**) by cross-sections of the body with $\sigma_{22}=const$, (**c**) by cross-sections of the body with $\sigma_{11}=const$.

At the ETH Polytechnic in Zurich, a test program for walls made of concrete, hollow masonry elements ZSW1-ZSW12 was also carried out [37]. The results of the experimental tests are given in Table 2, and in Figure 6. Figure 6a shows failure surface of the criterion with the experimental results of [37]. Figure 6b,c shows cross-sections with planes of the constant normal stress. A position of the yield stress from experimental tests are marked with diamonds.

The criterion surfaces are determined by the following parameters [MPa]: for a tension regime—$Y_{t1}=0.01$, $Y_{t2}=0.01$, $Y_{c1}=11.52$, $Y_{c2}=18.42$, $k_{t}=0.01$, $Y_{tt}=0.01$ and for a compression regime—$Y_{t1}=2.88$, $Y_{t2}=4.61$, $Y_{c1}=5.76$, $Y_{c2}=9.21$, $k_{c}=3.98$, $Y_{cc}=6.36$. The anisotropy ratio of the compressive strength is $Y_{c2}/Y_{c1}=1.60$. The material has almost zero tensile strength. As before, the load vector length was determined for each limit point as $\sqrt{\sigma_{11}^{2}+\sigma_{22}^{2}+2\sigma_{12}^{2}}$. The maximum difference of vectors resulting from the experiment data and those of the initial surface compared criterion does not exceed 7 percentage points, which shows a very good agreement of the proposed model with the experiment results.

## 4. The Constitutive Relations and Implementation of the Model

**Figure 7:**
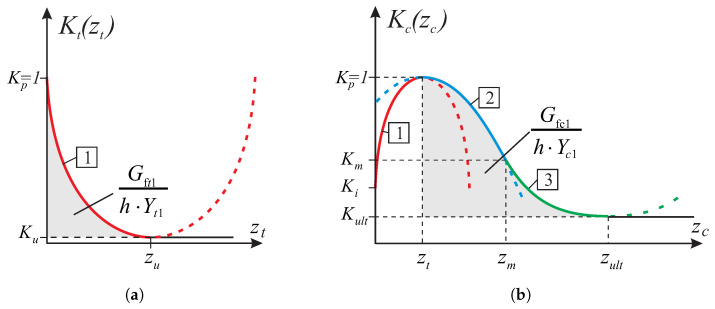
Degradation of material strength parameters curves adopted in the model: (**a**) tension regime, (**b**) compression regime.

The elastic-plastic orthotropic material is considered assuming an additive decomposition of the strain tensor into the elastic part $\varepsilon_{e}$ and the plastic part $\varepsilon_{p}$. The elastic part is defined by orthotropic Hooke’s law. The plastic part of the strain tensor is defined by a flow rule associated with the yield function given by the plasticity (failure) criterion written based on the forms (10) and (17) as:

(18) $$f_{\alpha }\left(\sigma ,z_{\alpha }\right)=\frac{1}{2}\sigma \cdot P_{\alpha }\cdot \sigma +p_{\alpha }\cdot \sigma -K_{\alpha }\left(z_{\alpha }\right)=0,$$

where the $K_{\alpha }\left(z_{\alpha }\right)$ are given functions with the real functional value from a closed interval [0, 1] that describes the type of hardening/softening (Figure 7), $z_{\alpha }$ are internal scalar hardening variables. The softening behavior is modeled with a smeared approach, where the localized damage is represented by the scalar, which is related by an equivalent length *h* to the released energy per unit cracked area, $G_{f}$. The length *h* should correspond to a dimension of the finite element mesh. As one can see in Figure 7, different fracture energies are introduced in the model, as additional parameters—the tensile fracture energy $G_{ft}$ and the compressive energy $G_{fc}$.

In the frame of reference coinciding with the orthotropy axes, we have the following matrix representations of tensors $P_{\alpha }$ and $p_{\alpha }$ in the Voigt notations for the plane stress $\sigma \Rightarrow {\left[\sigma_{11}\;\sigma_{22}\;\sqrt{2}\sigma_{12}\;\right]}^{T},$ (α=t for tension surface, α=c for compression surface):

(19) $$P_{\alpha }\Rightarrow P_{\alpha }=\left[\begin{array}{ccc} 2b_{11}^{\alpha } & 2b_{12}^{\alpha } & 0\\ 2b_{21}^{\alpha } & 2b_{22}^{\alpha } & 0\\ 0 & 0 & 4c^{\alpha } \end{array}\right],\;\;p_{\alpha }\Rightarrow p_{\alpha }=\left[\begin{array}{ccc} a_{1}^{\alpha } & a_{2}^{\alpha } & 0 \end{array}\right].$$

For composite criteria, the subscript α also denotes the number of the active surface. Let us first assume that only one surface is active, which will allow this marking to be omitted. In the framework of the mathematical theory of the elastic-plastic material a permissible stress state is any state of stresses for which f≤0. A stress state is called the elastic stress state if f<0. A plastic state refers to a stress state at the boundaries of the current elastic region for which f=0. The plastic part of the strain tensor is defined by a flow rule associated with the yield function given by the Equation (18). The flow rule defines the sign (direction) of plastic-strain increment in the following form:

(20) $${\dot{\varepsilon }}_{p}=\gamma \;{\left.\frac{\partial f\left(\sigma ,z\right)}{\partial \sigma }\right|}_{\sigma =\sigma^{T}}=\gamma \;\left(P\cdot \sigma +p\right)\equiv \gamma \;r,$$

where γ>0 is a plastic multiplier. After applying differentiation to orthotropic Hooke’s elastic law with the respect to the time and after substituting Equation (20) we obtain:

(21) $$\dot{\sigma }=C\cdot \left(\dot{\varepsilon }-\gamma \;r\right)\equiv C_{ep}\cdot \dot{\varepsilon },$$

where the elasto-plastic tangent operator $C_{ep}$ can be calculated after the parameter γ is known. Assuming that

(22) $$\dot{z}=\gamma \left(r\cdot r\right)\equiv \gamma {∥r∥}^{2}.$$

one can compute from the consistency condition

(23) $$\gamma \dot{f}\left(\sigma ,z\right)=0,\;\gamma >0,$$

the plastic multiplier

(24) $$\gamma =\frac{\langle r\cdot C\cdot \dot{\varepsilon }\rangle }{r\cdot C\cdot r+\partial K/\partial z\;{∥r∥}^{2}},$$

and the operator $C_{ep}$ in the following form:

(25) $$C_{ep}=C-\frac{\left(C\cdot r\right)⊗\left(r\cdot C\right)}{r\cdot C\cdot r+\partial K/\partial z\;{∥r∥}^{2}}\;.$$

The double symmetric fourth-order tensor of elastic material constants C in the orthotropic Hooke’s law can be conveniently defined by the compliance tensor $S\equiv C^{-1}$, which in the frame of reference aligned with the orthotropic axes can be written in Voigt notation for the plane stress as

(26) $$S\to \left[\begin{array}{ccc} \frac{1}{E_{1}} & -\frac{\nu_{21}}{E_{2}} & 0\\ -\frac{\nu_{12}}{E_{1}} & \frac{1}{E_{2}} & 0\\ 0 & 0 & \frac{1}{G_{12}} \end{array}\right].$$

where we have five technical in-plane moduli: $E_{1},\;E_{2}$ are Young’s moduli, $G_{12}$ is the shear modulus and $\nu_{12},\;\nu_{21}$ are Poisson’s ratios ($\nu_{12}/E_{1}=\nu_{21}/E_{2}$). It should be noted that two yield criteria are combined in the model into a composite yield surface and the intersection of different yield surfaces defines corners that require special attention in a numerical algorithm according to Koiter’s generalization.

### 4.1. Implementation into Finite Elements under the Plane Stress Condition

In this subsection, according to the convention adopted in many finite element programs the components of a symmetric second-order tensor are presented as a single column array, whereas fourth-order tensors are presented as two-dimensional arrays. The matrix representations of the tensors are shown in terms of the Cartesian components in the frame coinciding with the materialaxes of orthotropy.

The constitutive relationship in (21) is in the form of the “highly non-linear” differential equation which can be solved by the modified Euler method (usually the implicit Euler backward algorithm). Therefore, it is replaced by the incremental equation of the form:

(27) $$\Delta \sigma =C\;\left(\Delta \varepsilon -\gamma \;\tilde{r}\right)\equiv {\tilde{C}}_{ep}\;\Delta \varepsilon ,$$

where ${\tilde{C}}_{ep}$ is called the operator consistent with the integration algorithm of constitutive relations. We assume that for each $t_{n}\in \left[0,T\right]$ the strain increment $\Delta \varepsilon =\varepsilon_{n+1}-\varepsilon_{n}$ is known, thus the problem is strain driven, and we want to compute the stress state $\sigma_{n+1}$ for $t_{n+1}$. We assume:

(28) $$\sigma_{n+1}=\sigma_{n+1}^{trl}-\Delta \gamma \;Cr^{trl},$$

where $\sigma_{n+1}^{trl}=C\;\varepsilon_{n+1}$ is called the trial stress state and $r^{trl}$ is the gradient of $f\left(\sigma_{n+1}^{trl},z_{n}\right)$. The calculation of the multiplier Δγ>0 and the tensor function $\tilde{r}$ (Equation (27)) is significantly dependent on the realization of $\sigma_{n+1}^{trl}$. A detailed description of the numerical implementation into the FEM is given in [38] for the elastic-plastic material with the yield criteria of Huber–Mises–Hencky (isotropy) and Hill (orthotropy). The most important step is to calculate the multiplier using the quadratic equation of the variable Δγ. This step is significantly different from the case of the Hill yield criterion.

Based on the consistency condition (23) in the algorithmic form:

(29) $$f\left(\sigma_{n+1}^{T},z_{n+1}\right)=\frac{1}{2}\sigma_{n+1}^{T}P\;\sigma_{n+1}+p^{T}\;\sigma_{n+1}-K\left(z_{n+1}\right)=0,$$

we obtain after the substitution of (28) into (29) a following quadratic equation (indices n+1 suppressed):

(30) $$A\;\Delta \gamma^{2}+B\;\Delta \gamma +C=0,$$

where:

(31) $$\begin{array}{c} A=\frac{1}{2}{\left(C\;r_{n+1}^{trl}\right)}^{T}P\;C\;r_{n+1}^{trl}-\frac{\partial^{2}K}{\partial z^{2}}\;{\left[{\left(r_{n+1}^{trl}\right)}^{T}r_{n+1}^{trl}\right]}^{2},\\ B=-\left[\frac{1}{2}\sigma_{n+1}^{trl}P\;Cr_{n+1}^{trl}+\frac{1}{2}{\left(C\;r_{n+1}^{trl}\right)}^{T}P\;\sigma_{n+1}^{trl}+p^{T}C\;r_{n+1}^{trl}+\frac{\partial K}{\partial z}\;{\left(r_{n+1}^{trl}\right)}^{T}r_{n+1}^{trl}\right],\\ C=f^{trl}\left(\sigma_{n+1}^{trl},z_{n}\right). \end{array}$$

The solution of the Equation (30) is particularly simple if the hardening/softening function $K\left(z\right)$ is the second degree polynomial of the form as shown in the Figure 7. The last part of (30) may become then equal to the yield function of the trial state $f^{trl}$. It can be seen that the linearization of the Equation (30) does not lead to significant errors. Determining the plastic multiplier from the linear part of Equation (30) in the form Δγ=−B/C does not lead to large errors, although it depends on the assumed strain increment step length.

A model based on two criteria complicates the procedure algorithm. If only one of the criteria is exceeded, e.g., the stresses are reduced to the exceeded surface according to the algorithm (28)–(30), as for a model based on only one criterion. It remains, to establish which criterion is actually exceeded.

A separate case is exceeding both conditions at the same time:

$$f_{1}^{trl}>0\;\wedge \;f_{2}^{trl}>0.$$

This is the case where the point is outside the edge joining both surfaces and requires a different procedure. A linear combination of gradients has been used here (compare Koiter’s law). The component of the plastic multiplier is calculated separately for the tensile criterion and for the compression criterion. Plastic strains and stress state components at time $t_{n+1}$ can be determined on the basis of the formulas:

(32) $$\begin{array}{c} \varepsilon_{n+1}^{p}=\varepsilon_{n}^{p}+\Delta \gamma_{1}\;r_{n+1}^{1,trl}+\Delta \gamma_{2}\;r_{n+1}^{2,trl},\\ \sigma_{n+1}=\sigma_{n+1}^{trl}-\Delta \gamma_{1}\;C\;r_{n+1}^{1,trl}-\Delta \gamma_{2}\;C\;r_{n+1}^{2,trl},\\ C_{ep}=C-\frac{C\;r_{n+1}^{1,trl}{\left(r_{n+1}^{1,trl}\right)}^{T}C^{T}}{{\left(r_{n+1}^{1,trl}\right)}^{T}C\;r_{n+1}^{1,trl}+\partial K_{1}/\partial z_{1}\;{∥r_{n+1}^{1,trl}∥}^{2}}\;-\frac{C\;r_{n+1}^{2,trl}{\left(r_{n+1}^{2,trl}\right)}^{T}C^{T}}{{\left(r_{n+1}^{2,trl}\right)}^{T}C\;r_{n+1}^{2,trl}+\partial K_{2}/\partial z_{2}\;{∥r_{n+1}^{2,trl}∥}^{2}}\;. \end{array}$$

After similar transformations as before, the following system of quadratic equations is obtained.

(33) $$\left\{\begin{array}{c} A_{1}{\left(\Delta \gamma_{1}\right)}^{2}+B_{1}\Delta \gamma_{1}+C_{1}+D_{1}{\left(\Delta \gamma_{2}\right)}^{2}+E_{1}\Delta \gamma_{2}+F_{1}\Delta \gamma_{1}\Delta \gamma_{2}=0\\ A_{2}{\left(\Delta \gamma_{2}\right)}^{2}+B_{2}\Delta \gamma_{2}+C_{2}+D_{2}{\left(\Delta \gamma_{1}\right)}^{2}+E_{2}\Delta \gamma_{1}+F_{2}\Delta \gamma_{1}\Delta \gamma_{2}=0 \end{array}\right.,$$

where some of the coefficients are analogous to the problem with one active surface and are marked with letters $A_{\alpha },B_{\alpha },C_{\alpha }$ (α=1,2):

(34) $$\begin{array}{cc} A_{\alpha } & =\frac{1}{2}{\left(Cr_{n+1}^{\alpha ,trl}\right)}^{T}P_{\alpha }Cr_{n+1}^{\alpha ,trl}-K_{\alpha ,n}^{\prime \prime }{\left[{\left(r_{n+1}^{\alpha ,trl}\right)}^{T}r_{n+1}^{\alpha ,trl}\right]}^{2},\\ B_{\alpha } & =-\frac{1}{2}\sigma_{n+1}^{trl}P_{\alpha }Cr_{n+1}^{\alpha ,trl}-\frac{1}{2}{\left(Cr_{n+1}^{\alpha ,trl}\right)}^{T}{P_{\alpha }\sigma }_{n+1}^{trl}-{\left(p_{\alpha }\right)}^{T}Cr_{n+1}^{\alpha ,trl}-K_{\alpha ,n}^{\prime }{\left(r_{n+1}^{\alpha ,trl}\right)}^{T}r_{n+1}^{\alpha ,trl},\\ C_{\alpha } & =f_{\alpha }^{trl}\left(\sigma_{n+1}^{trl},z_{n}^{\alpha }\right). \end{array}$$

The remaining coefficients depend on the data related to both boundary surfaces and the formulas can be expressed as follows:

(35) $$\begin{array}{cc} D_{1} & =\frac{1}{2}{\left(C\;r_{n+1}^{2,trl}\right)}^{T}P_{1}C\;r_{n+1}^{2,trl},\;\;D_{2}=\frac{1}{2}{\left(C\;r_{n+1}^{1,trl}\right)}^{T}P_{2}C\;r_{n+1}^{1,trl},\\ E_{1} & =-\frac{1}{2}\sigma_{n+1}^{trl}P_{1}C\;r_{n+1}^{2,trl}-\frac{1}{2}{\left(C\;r_{n+1}^{2,trl}\right)}^{T}P_{1}\sigma_{n+1}^{trl}-p_{1}^{T}C\;r_{n+1}^{2,trl},\\ E_{2} & =-\frac{1}{2}\sigma_{n+1}^{trl}P_{2}C\;r_{n+1}^{1,trl}-\frac{1}{2}{\left(C\;r_{n+1}^{1,trl}\right)}^{T}P_{2}\sigma_{n+1}^{trl}-p_{2}^{T}C\;r_{n+1}^{1,trl},\\ F_{1} & ={\left(C\;r_{n+1}^{1,trl}\right)}^{T}P_{1}C\;r_{n+1}^{2,trl},\;\;\;F_{2}={\left(C\;r_{n+1}^{2,trl}\right)}^{T}P_{2}C\;r_{n+1}^{1,trl}. \end{array}$$

**Figure 8:**
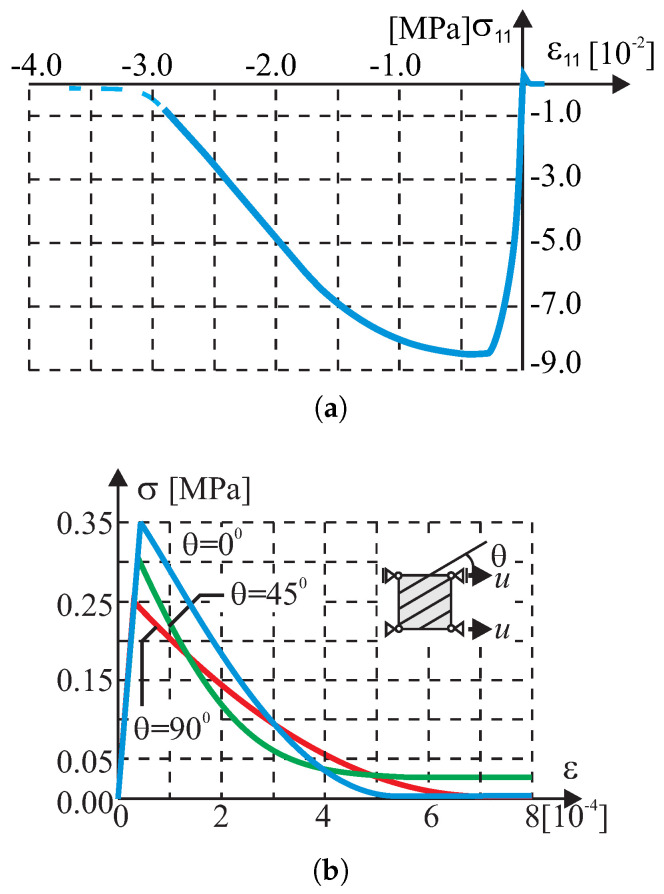
Single-element test results. Relationship σ−ε: (**a**) compression along 1(x) axis, (**b**) tensile softening in the direction rotated by an angle θ to the first orthotropy axis.

**Figure 9:**
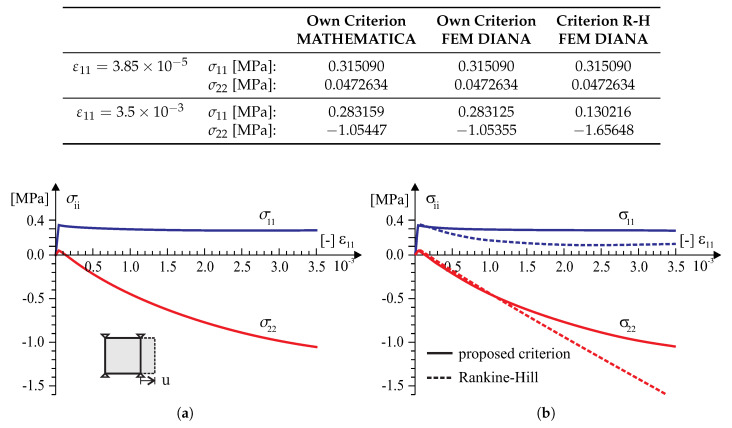
A homogeneous strain field $\varepsilon_{11}>0$ and $\varepsilon_{22}=0$ single element test results: (**a**) symbolic computation by MATHEMATICA [40], (**b**) finite element method (FEM) analysis in TNO DIANA [39].

**Figure 10:**
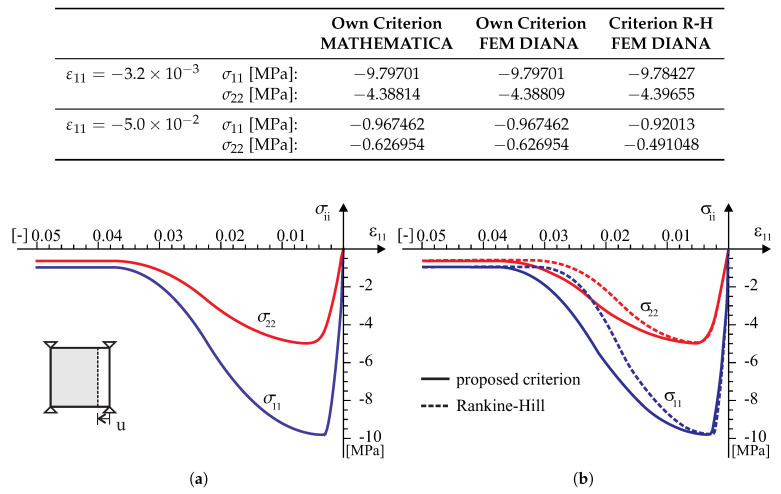
A homogeneous strain field $\varepsilon_{11}<0$ and $\varepsilon_{22}=0$ single element test results: (**a**) symbolic computation by MATHEMATICA [40], (**b**) FEM analysis in TNO DIANA [39].

**Figure 11:**
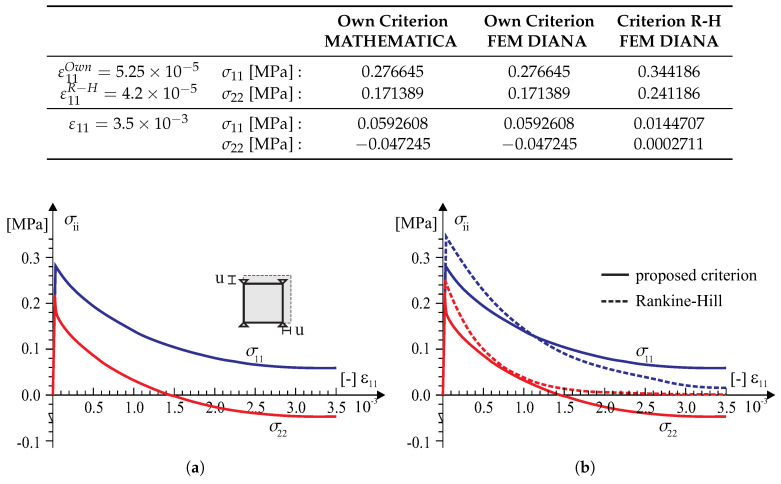
A strain field $\varepsilon_{11}>0$ and $\varepsilon_{22}>0$ single element test results: (**a**) symbolic computation by MATHEMATICA [40], (**b**) FEM analysis in TNO DIANA [39].

**Figure 12:**
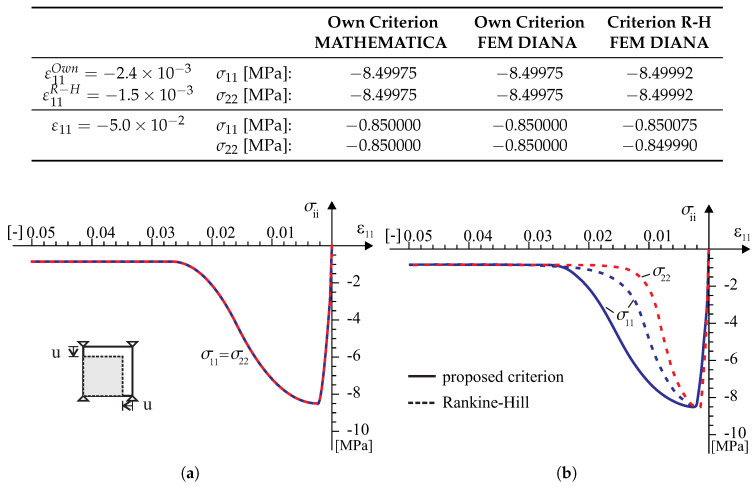
A strain field $\varepsilon_{11}<0$ and $\varepsilon_{22}<0$ single element test results: (**a**) symbolic computation by MATHEMATICA [40], (**b**) FEM analysis in TNO DIANA [39].

According to the above numerical algorithm, the several models with the failure criterion of the type (18) has been coded in the programming language FORTRAN and next implemented into a commercial finite element code DIANA [39]. There are standard Newton–Raphson and Riks algorithms for the solving nonlinear equilibrium equations in the program. Because of this, implementation of the model into a finite element code is done at the integration point level by means of user-defined subroutine USRMAT. The subroutine lets the user specify a general nonlinear material behavior by updating the state variables over the equilibrium step n→n+1 within a framework of an incremental–iterative algorithm of finite element method. Both the return-mapping algorithm allowing the stresses to be returned to the yield surface and a consistent tangent stiffness operator have been coded. The implementation of the model is presented both at the single element tests (see Figure 8, Figure 9, Figure 10, Figure 11 and Figure 12) and at the structural level test (next section).

### 4.2. Results of the Single-Element Tests

Tests in the plane stress were performed in the homogeneous stress state and with one isoparametric continuum finite element (four-node, linear interpolation and Gaussian integration). The displacement-controlled load diagram is shown in Figure 8. The following material data are adopted: the moduli of elasticity in two directions of orthotropy: $E_{1}$ = $E_{2}$ = 8 [GPa], the shear modulus: $G_{12}=3.478$ [GPa], the Poissonś coefficient: $\nu_{12}=0.15$, that is, as for the isotropic material. The strength parameters associated with the initial tensile failure surface of the criterion are: $Y_{c1}=17$, $Y_{c}2=17$, $Y_{t1}=0.35$, $Y_{t2}=0.25$, $Y_{tt}=0.22$, $k_{12}=0.296$ [MPa]. The strength parameters associated with the compressive surface are: $Y_{c1}=8.5$, $Y_{c2}=8.5$, $Y_{t1}=8.5$, $Y_{t2}=8.5$, $Y_{tt}=8.5$, $k_{12}=4.9$ [MPa]. The material parameters are typical for unreinforced walls in terms of the values and the proportion between them. The strength degradation curves are adopted with two internal scalar parameters: $z_{t}$ for the tensile softening and $z_{c}$ for the softening during compression (Figure 7). The curves are matched to set the fracture energies along the first orthotropic material axis as $G_{f1}=54.0$ [J/m${}^{2}$] during tension and as $G_{fc1}=20.0$ [kJ/m${}^{2}$] during compression.

In the test with the homogeneous deformation field, all eight degrees of freedom in the four-node finite element were fixed to force the desired linear deformation. To compare the behavior of our model in the tests, the model of the Rankine–Hill criterion was used, which is standard in the DIANA system and dedicated to the analysis of masonry structures. The following parameters of the Rankine–Hill model are adopted: $Y_{c1}=8.5Y$, $Y_{c2}=8.5Y$, β = −1.0, γ=3.0, $Y_{t1}=0.35Y$, $Y_{t2}=0.25Y$, α=1.0, where Y=1 [MPa] and the tensile fracture energies $G_{fX}=54.0$ [J/m${}^{2}$], $G_{fY}=18.0$ [J/m${}^{2}$] and the compressive fracture energies $G_{fcX}=20.0$ [kJ/m${}^{2}$] and $G_{fcY}=15.0$ [kJ/m${}^{2}$]. The algorithm of the own model was also programmed in MATHEMATICA. As a result, it was possible to control the correctness of the algorithm and its implementation in both computing environments. Results of the tests are presented in Figure 9, Figure 10, Figure 11 and Figure 12.

## 5. Tests at the Structural Level

Two examples of the proposed constitutive model are briefly presented here as the tests at the structural level. More information on the tests can be found in the works [41,42].

**Figure 13:**
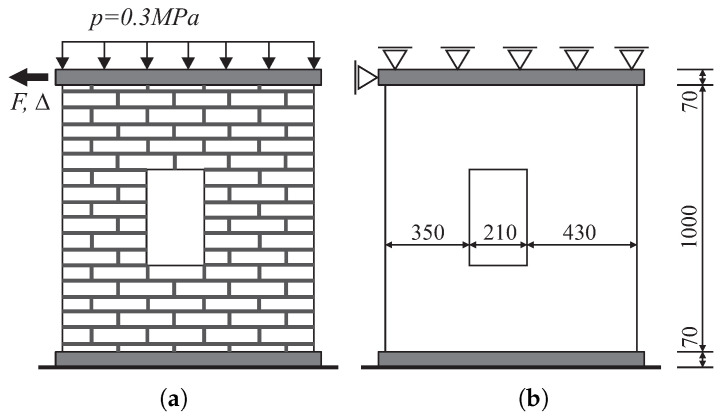
Walls tested by Raijmakekers and Vermeltfoort [43]: (**a**) load scheme for shear walls, (**b**) geometry and static sheme for numerical analysis.

**Figure 14:**
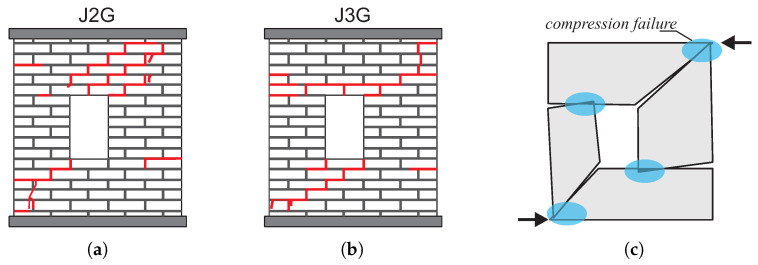
Experimental crack pattern at failure load [43] (**a**,**b**). (**c**) Mechanism of destruction.

The first example is restricted to the numerical simulation of the load capacity tests of masonry structures in the plane stress state that were conducted experimentally in [43]. In laboratory tests, two similar wall fragments marked as J2G and J3G were tested, Figure 13. The masonry shear walls with an opening and the thickness of 10 mm were built from 18 courses of masonry cement-sand units 210 × 52 × 100 [mm]. The top and bottom courses were fully clamped in steel beams. The wall is shearing with the horizontal force *F* under displacement control. The top edge can move with a horizontal displacement (Figure 13). The wall was first vertically loaded through a steel beam to the value *p* = 0.3 kN/m${}^{2}$ that remains constant through the subsequent loading steps of the horizontal force up to the maximum horizontal displacement of Δ = 16 mm. The beam movement was limited to the horizontal direction, i.e., the lower and upper edges of the wall were kept parallel. Figure 14 shows the forms of cracks obtained in the tests.

Cracks appeared diagonally between the opening and the lower and upper corners of the wall. In addition, tensile cracks appeared on the outer vertical edges of the wall, on both sides of the opening at the top of the left pillar and at the bottom of the right pillar, and their development ran from the outside to the center of the wall. The failure mechanism resulting from the wall tests is shown schematically in the Figure 14. As can be seen, the kinematics of the system focuses on the movement of four blocks connected by hinges. Due to the development of the material crushing zone, also marked in the drawing, the mechanism will activate the compression criterion.

For the numerical simulation of the wall failure using the model based on the own criterion, a 1989 mesh of four-node flat finite elements with an average side length of 23 [mm] was built. The values of material parameters used in the numerical simulation are shown in the Table 3. The values given in the table were adopted on the basis of work [44], where the data were adopted based on the results of experimental tests [43], and the supplementary parameters result from the homogenization procedures and were taken from work [16].

**Figure 15:**
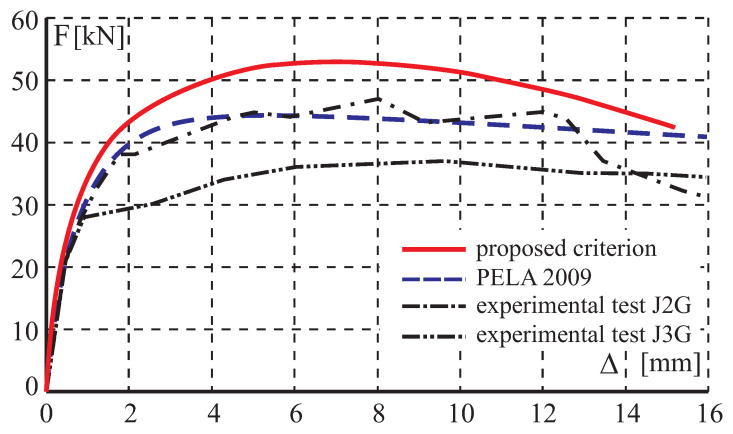
Horizontal load–horizontal displacement diagrams.

**Figure 16:**
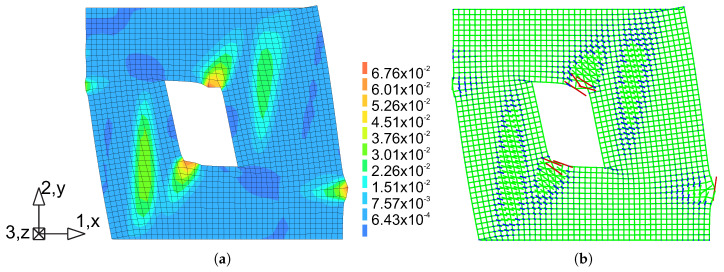
Maps of maximum principal plastic strain at Δ = 12 mm: (**a**) crack (tensile) zones, (**b**) directions of the tensile strain.

**Figure 17:**
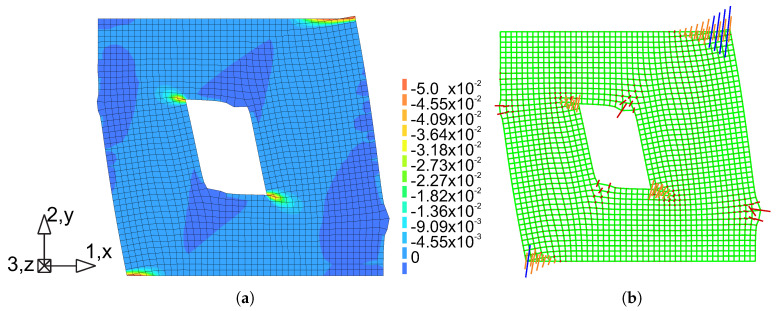
Maps of minimum principal plastic strain at Δ = 12 mm: (**a**) compressive failure zones, (**b**) directions of the compressive strain.

The comparison between numerical and experimental load–displacement diagrams, for wall J2G and J3G, is given in Figure 15. Apart from own calculation, results obtained by Pelá in [44] are also shown. Good agreement is found in the elastic range and satisfactory agreement in the inelastic range, although slightly worse in terms of the load capacity than the results obtained in [44]. The maximum divergence is around 17%. It is possible to better match the results of the experiment, however it requires additional calculations and time-consuming calibration of strength parameters and fracture energies. The behavior of the wall at the horizontal displacement Δ=12 mm is depicted in Figure 16 and Figure 17 in terms of the maximum and minimum principal plastic strains, respectively. Both tensile and compressive failure zones are captured by the model. This indicates that the wall deformability and the general mechanism of its destruction are correctly reflected.

Figure 16 shows the principal plastic strains $\varepsilon_{1}^{p}$ obtained from own simulations. They can be interpreted as the distribution of material failure zones as a result of exceeding the criterion in tension. These are also zones of development of cracks in the structure. Figure 16a shows the strain results in the form of maps plotted on a deformed finite element mesh. For a better effect, the deformation has been scaled 10 times.

In Figure 16b, the results in the vector form were superimposed on the mesh, wich allows to compare the length of the strain and the direction. Figure 17a shows the principal plastic deformations $\varepsilon_{3}^{p}$, which in Figure 17b are shown in vector form. Areas with large $\varepsilon_{3}^{p}$ strains can be interpreted as zones of material failure due to possible material crushing.

**Figure 18:**
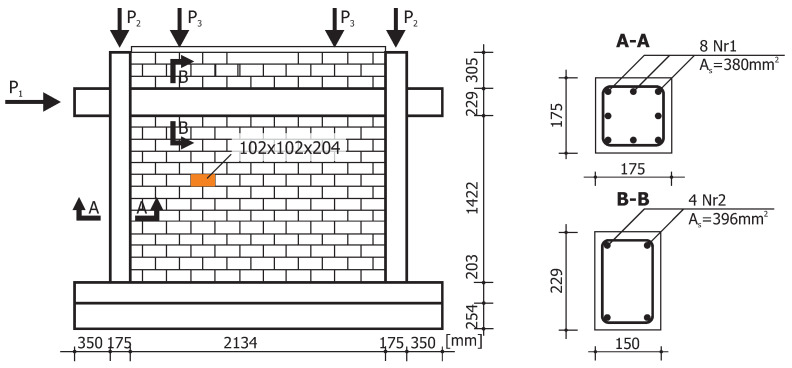
Geometry and loads for masonry infill wall tested in [45].

In the second example, the behavior of the masonry infill wall that is built within a reinforced concrete frame is numerically simulated (Figure 18). The wall was experimentally tested in [45]. The frame and the wall were first subjected to the vertical loads $P_{2}=97.8$ kN and $P_{3}=48.9$ kN that remain constant through the subsequent loading steps of the horizontal force $P_{1}$ up to the failure.

**Figure 19:**
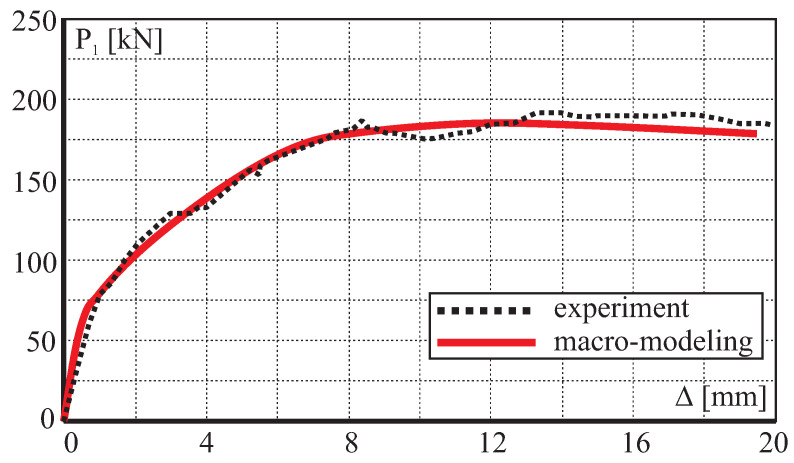
Comparison of load–displacement diagram.

For the numerical analyses 4-noded quadrilaterals, linear plane stress, and continuum elements are utilized. The subsequent loading steps of the horizontal force up to the maximum horizontal displacement Δ = 20 mm were analyzed under a displacement control. The material properties of the model are given in Table 4. The comparison between numerical and experimental load–displacement diagrams is given in Figure 19. The low initial vertical load combined with the confinement provided by the reinforced concrete frame yields extremely ductile behavior.

**Figure 20:**
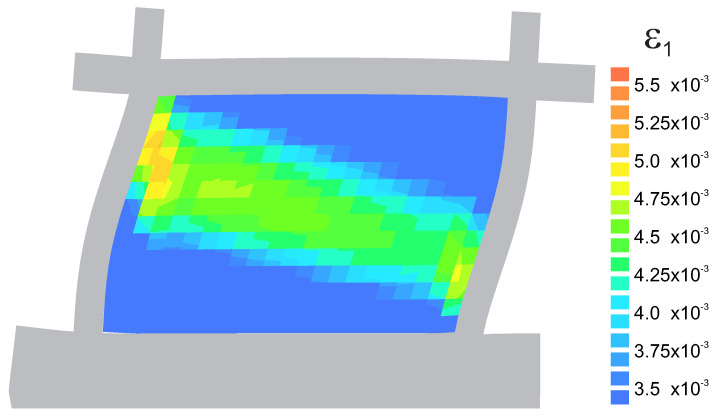
Failure mechanism obtained by numerical simulation as map of maximum principal plastic strain at Δ=10 mm.

In Figure 20, a crack zone is plotted as the map of the tensile principal plastic strain. A good agreement is found with respect to the calculated collapse load value. The model gives the response that is close to the experimental equilibrium path. At the ultimate stage, a well-defined failure mechanism is formed with a final diagonal shear band going from one corner of the specimen to the other.

The precise localization of cracks can also be achieved using models based on a smeared crack approach [46,47], fracture-based models with advanced crack tracking techniques [44] or by using micro-modeling [16]. To the evaluation of historic buildings for dynamic actions methods based on ultimate load-bearing capacity are used. Especially in combination with the monitoring based on operational modal analysis (OMA) techniques, it is becoming a popular practice [48]. An alternative may also be the relatively young topic of multi-scale modeling [49] or to describe diffuse cracks a gradient-enhanced damage model based on nonlocal displacements and the extended finite element method (XFEM) for sharp cracks [50,51]. However, if we compare the numerical result presented here with the failure mechanisms shown in Figure 16 and Figure 20, we find a good agreement and confirm the ability of our own model to correctly reflect the behavior of the structure.

## 6. Conclusions

The mechanical behavior of the continuum material model can be described using constitutive relations based on the theory of tensor functions. This theory, together with the theorems on the representation of tensor functions, constitute an effective mathematical tool for the formulation of constitutive relations of the orthotropic material. The invariance requirements of the isotropy of space and the orthotropic symmetry of materials are easily considered, so that the orientation of the material in space does not affect its constitutive relation. It is possible to obtain the analytical transparency as well as to maintain the required universalism of constitutive equations, see, e.g., [30]. The composite orthotropic failure criterion of the proposed model is constructed from two square scalar functions that are dependent on three orthotropic invariants of the plane stress tensor. In general, the criterion needs to have the twelve material parameters to define the failure surface. However, in practice, only seven of them are used that are obtained from the appropriate uniaxial, biaxial and shear strength tests. The criterion is an example of the orthotropic failure criteria, which can be treated as a generalization of the well-known Hoffman failure criterion that is often used for brittle orthotropic materials in which compressive strengths and tensile strengths are significantly different.

The numerical tests confirm correctness of the implementation and the ability of the models to reproduce failure modes in the structural tests in certain situations. They also show that it is possible to incorporate strain softening into the proposed class of models with a single yield surface. At present, the implementation of the other models within the framework of multisurface plasticity is being tested. Although for the multisurface plasticity the intersection of the different yield surfaces defines corners that require special attention in a numerical algorithm, it has the advantage of engaging different hardening laws for each surface, which might be more physically realistic due to the distinction of tension and compression regimes. The choice of a hardening parameter is not crucial because the available experimental data are scarce.

The commercial version of Diana with the so-called user-supplied subroutine mechanism can be used as the development environment for computational materials research. We have used the user-supplied subroutine USRMAT to implement new material models for continuum spatial and plane stress elements. The new models can be applied to a variety of structural problems, to single element tests but also to simulate physical experiments, using different elements types and using standard features of the program such as advanced solution procedures, for instance indirect displacement control with full Newton–Raphson. Also, the use of the post-processing capabilities of the program is an advantage, although the post-processing of the user-defined status variables might be more user-friendly.

## Figures and Tables

**Table 1:** Comparison of the proposed criterion with the experimental results of [36].

			Experimental			Numerical			
Panel	$\sigma_{1}/\sigma_{2}$	ϕ	[MPa]			[MPa]			Exp/Num
		[°]	$\sigma_{11}$	$\sigma_{22}$	$\tau_{12}$	$\sigma_{11}$	$\sigma_{22}$	$\tau_{12}$	[-]
K1	−0.09	22.5	−0.08	−0.92	0.42	−0.09	−1.04	0.47	0.88
K2	−0.05	22.5	−0.17	−1.42	0.62	−0.17	−1.35	0.59	1.05
K3	0.0	0.0	0.00	−7.63	0.00	0.00	−7.61	0.00	1.01
K4	0.0	90.0	−1.83	0.00	0.00	−1.87	0.00	0.00	0.98
K6	0.0	45.0	−0.32	−0.32	0.32	−0.43	−0.43	0.43	0.74
K7	0.0	22.5	−0.39	−2.25	0.93	−0.37	−2.15	0.89	1.05
K8	0.0	67.5	−0.22	−0.04	0.09	−0.22	−0.04	0.08	1.01
K10	0.33	0.0	−2.11	−6.44	0.00	−2.02	−6.15	0.00	1.05
K11	0.31	22.5	−2.04	−4.49	1.23	−2.02	−4.46	1.22	1.01
K12	0.30	45.0	−2.03	−2.03	1.08	−2.01	−2.01	1.07	1.01

**Table 2:** Comparison of the proposed criterion with the experimental results of [37].

			Experimental			Calculated			
Panel	σ1σ1σ2σ2	ϕ	[MPa]			[MPa]			Exp/Num
		[°]	$\sigma_{11}$	$\sigma_{22}$	$\tau_{12}$	$\sigma_{11}$	$\sigma_{22}$	$\tau_{12}$	[-]
ZSW1	0.0	0.0	0.00	−9.12	0.00	0.00	−9.21	0.00	0.99
ZSW2	0.14	0.0	−6.12	−0.83	0.00	−6.05	−0.82	0.00	1.01
ZSW4	1.53	0.0	−5.98	−9.13	0.00	−5.58	−8.52	0.00	1.07
ZSW5	0.0	45.0	−3.06	−3.06	3.06	−3.06	−3.06	3.06	1.00
ZSW6	0.22	45.0	−4.60	−4.60	2.93	−4.69	−4.69	2.98	0.98
ZSW7	1.0	45.0	−6.12	−6.12	0.00	−6.36	−6.36	0.00	0.96
ZSW8	0.0	67.5	−2.34	−0.40	0.97	−2.34	−0.40	0.97	1.00
ZSW9	0.0	22.5	−0.97	−5.66	2.35	−0.97	−5.66	2.35	1.00

**Table 3:** Masonry shear wall. Material properties of the model.

Elastic Moduli						
	$E_{11}$	$E_{22}$	$\nu_{12}$	$G_{12}$		
	[MPa]	[MPa]	−	[MPa]		
	7520	3960	0.09	1460		
**Uniaxial, Biaxial and Shear Strengths in the Orthotropic Axes [MPa]**						
	$Y_{c1}^{\alpha }$	$Y_{c2}^{\alpha }$	$Y_{t1}^{\alpha }$	$Y_{t2}^{\alpha }$	$Y_{\alpha \alpha }$	$k_{12}$
Compression α=c	5.25	3.75	2.625	1.825	3.0	0.45
Tension α=t	11.0	7.5	0.35	0.25	0.30	0.30
Fracture energies in J/m${}^{2}$		$G_{fc1}$ = 2350		$G_{ft1}$ = 43.3		

**Table 4:** Characteristics of materials for orthotropic model of masonry.

Elastic Moduli						
	$E_{11}$, MPa	$E_{22}$, MPa	$\nu_{12}=\nu_{21}$	$G_{12}$		
	[MPa]	[MPa]	[-]	[MPa]		
	13,790	13,790	0.16	3480		
**Characteristics of the Strength Surface in MPa**						
	$Y_{c1}^{\alpha }$	$Y_{c2}^{\alpha }$	$Y_{t1}^{\alpha }$	$Y_{t2}^{\alpha }$	$Y_{\alpha \alpha }$	$k_{12}^{\alpha }$
Compression (α=c)	20.7	20.7	20.7	20.7	23.00	5.0
Tension (α=c)	40.0	40.0	1.38	1.38	1.2	0.5
Fracture energies in J/m${}^{2}$		$G_{fc1}$ = 350		$G_{ft1}$ = 16.7		

## References

[1] Lourenço P. (2009). Recent advances in Masonry modelling: Micromodelling and homogenisation. Multiscale Model. Solid Mech. Comput. Approaches.

[2] Giamundo V., Sarhosis V., Lignola G., Sheng Y., Manfredi G. (2014). Evaluation of different computational modelling strategies for the analysis of low strength masonry structures. Eng. Struct..

[3] Lemos J. (2019). Discrete Element Modeling of the Seismic Behavior of Masonry Construction. Buildings.

[4] Asteris P., Moropoulou A., Skentou A., Apostolopoulou M., Mohebkhah A., Cavaleri L., Rodrigues H., Varum H. (2019). Stochastic Vulnerability Assessment of Masonry Structures: Concepts, Modeling and Restoration Aspects. Appl. Sci..

[5] Yokel F., Fattal S. (1976). Failure hypothesis for masonry shear walls. J. Struct. Div..

[6] Mann W., Muller H. (1982). Failure of shear-stressed masonry—An enlarged theory, tests and application to shear walls. Proc. Br. Ceram. Soc..

[7] Page A. (1981). The biaxial compressive strength of brick masonry. Proc. Inst. Civ. Eng..

[8] Page A. (1983). The strength of brick masonry under biaxial tension-compression. Int. J. Mason. Constr..

[9] Dhanasekar M., Page A., Kleeman P. (1985). The failure of brick masonry under biaxial stresses. Proc. Inst. Civ. Eng..

[10] Ganz H., Thürlimann B. Strength of brick walls under normal force and shear. Proceedings of the 8th International Symposium on Load Bearing Brickwork.

[11] Seim W. (1995). Nümerische Modellierung Des Anisotropen Versagens Zweiachsig Beanspruchter Mauerwerksscheiben. Ph.D. Thesis.

[12] Tsai S., Wu E. (1971). A general theory of strength for anisotropic materials. J. Compos. Mater..

[13] Syrmakezis C., Asteris P. (2001). Masonry failure criterion under biaxial stress state. J. Mater. Civ. Eng..

[14] Berto L., Saetta A., Scotta R., Vitaliani R. (2002). An orthotropic damage model for masonry structures. Int. J. Numer. Methods Eng..

[15] Hoffman O. (1967). The brittle strength of orthotropic materials. J. Compos. Mater..

[16] Lourenço P. (1996). Computational Strategies for Masonry Structures. Ph.D. Thesis.

[17] Lourenço P., Borst R., Rots J. (1997). Plane stress softening plasticity model for orthotropic materials. Int. J. Numer. Methods Eng..

[18] Małyszko L. (2005). Modelowanie Zniszczenia w Konstrukcjach Murowych z Uwzglȩdnieniem Anizotropii (Failure Modeling in Masonry Structures Taking into Account Anisotropy).

[19] Geniev G.A., Kurbatov A., Samedov F. (1993). Voprosy Procznosti I Plasticznosti Anizotropnyh Materialov.

[20] Lourenço P. (1997). An Anisotropic Macro-Model for Masonry Plates and Shells: Implementation and Validation.

[21] Jasiński R. (2017). Research and Modeling of Masonry Shear Walls. Ph.D. Thesis.

[22] Gambarotta L., Lagomarsino S. (1997). Damage models for the seismic response of brick masonry shear walls. Part II: The continuum model and its applications. Earthq. Eng. Struct..

[23] Massart T., Peerlings R., Geers M. (2004). Mesoscopic modeling of failure and damage-induced anisotropy in brick masonry. Eur. J. Mech. Solids.

[24] Milani G., Lourenço P., Tralli A. (2006). Homogenised limit analysis of masonry walls. Part I: Failure Surfaces. Comput. Struct..

[25] Noii N., Aldakheel F., Wick T., Wriggers P. (2020). An adaptive global-local approach for phase-field modeling of anisotropic brittle fracture. Comput. Methods Appl. Mech. Eng..

[26] Noii N., Khodadadian A., Wick T. (2020). Bayesian Inversion for Anisotropic Hydraulic Phase-Field Fracture. arXiv.

[27] Khodadadian A., Noii N., Parvizi M., Abbaszadeh M., Wick T., Heitzinger C. (2020). A Bayesian estimation method for variational phase-field fracture problems. Comput. Mech..

[28] Pelá L., Cervera M., Roca P. (2011). Continuum damage model for orthotropic materials: Application to masonry. Comput. Methods Appl. Mech. Eng..

[29] Małyszko L., Jemioło S., Bilko P. On the use of the Hoffman criterion in the continuum structural model for masonry panels. Proceedings of the 7th International Conference AMCM 2011.

[30] Małyszko L., Jemioło S., Gajewski M., Bilko P. (2009). FEM and Constitutive Modelling in Failure Analyses of Masonry Structures. Orthotropic Failure Criteria. WTA-Schriftenreihe.

[31] Wagner W., Klinkel S., Gruttmann F. (2002). Elastic and plastic analysis of thin-walled structures using improved hexahedral elements. Comput. Struct..

[32] Rolfes R., Vogler M., Czichon S., Ernst G. (2011). Exploiting the structural reserve of textile composite structures by progressive failure analysis using a new orthotropic failure criterion. Comput. Struct..

[33] Boehler J. (1987). Applications of Tensor Functions in Solid Mechanics.

[34] Jemioło S., Małyszko L. (2008). New failure criteria for orthotropic materials. Monograph Computer Systems Aided Science and Engineering Work in Transport, Mechanics and Electrical Engineering.

[35] Jemioło S., Gajewski M., Małyszko L., Bilko P. (2009). Orthotropic elastic-plastic model of masonry for numerical analysis in spatial stress state. Lightweight Structures in Civil Engineering, Contemporary Problems, 15th International Seminar of IASS PC.

[36] Ganz H., Thürlimann B. (1982). Tests on the Biaxial Strength of Masonry.

[37] Lurati F., Graf H., Thürlimann B. (1990). Experimental Determination of the Strength Parameters of Concrete Masonry.

[38] Simo J., Hughes T. (1998). Computational Inelasticity.

[39] DIANA (2009). User’s Manual Release 9.3.

[40] Wolfram (2010). Mathematica.

[41] Małyszko L., Bilko P. (2016). Symulacje numeryczne wyczerpania nośności ramy żelbetowej wypełnionej murem (Numerical simulations of bearnig capacity of reinforced concrete frame with infill masonry). Inżynieria I Bud..

[42] Klovanych S., Małyszko L. (2019). Plasticity in Structural Engineering.

[43] Raijmakers T.M.J. (1992). Deformation Controlled Tests in Masonry Shear Walls: Report B-92-1156.

[44] Pelá L. (2009). Continuum Damage Model for Nonlinear Analysis of Masonry Structures. Ph.D. Thesis.

[45] Mehrabi A.B., Shing P.B., Schuller M.P., Noland J.L. (1994). Performance of Masonry-Infilled R/C Frames under in-Plane Lateral Loads. CU/SR-94/6.

[46] Gattulli V., Lofrano E., Paolone A., Pirolli G. (2017). Performances of FRP reinforcements on masonry buildings evaluated by fragility curves. Comput. Struct..

[47] Ungermann J., Adam V. (2020). Fictitious Rough Crack Model (FRCM): A Smeared Crack Modelling Approach to Account for Aggregate Interlock and Mixed Mode Fracture of Plain Concrete. Materials.

[48] Milani G., Shehu R., Valente M. (2018). Simple Limit Analysis Approach for the Optimal Strengthening of Existing Masonry Towers.

[49] Qian Z., Schlangen E., Ye G., Breugel K. (2017). Modeling Framework for Fracture in Multiscale Cement-Based Material Structures. Materials.

[50] Meschke G., Dumstorff P. (2007). Energy-based modeling of cohesive and cohesionless cracks via X-FEM. Comput. Methods Appl. Mech. Eng..

[51] Tamayo-Mas E., Feliu-Fabá J., Casado-Antolin M., Rodríguez-Ferran A. (2019). A continuous-discontinuous model for crack branching. Int. J. Numer. Methods Eng..

